# THE GROWTH OF THE HOME CARE WORKFORCE FROM 2000 TO 2019 AND THE ROLE OF PUBLIC FINANCING

**DOI:** 10.1093/geroni/igad104.2039

**Published:** 2023-12-21

**Authors:** Karen Shen, Jeromie Ballreich, Chanee Fabius, Katherine Ornstein, Jennifer Reckrey

**Affiliations:** Johns Hopkins Bloomberg School of Public Health, Baltimore, Maryland, United States; Johns Hopkins Bloomberg School of public health, Baltimore, Maryland, United States; Johns Hopkins University, Baltimore, Maryland, United States; Johns Hopkins University, Baltimore, Maryland, United States; Icahn School of Medicine at Mount Sinai, New York City, New York, United States

## Abstract

Between 2000 and 2019, employment in home care industries grew from under one million workers to more than 3.5 million workers in 2019. However, there is significant variation across states in this growth, with some states experiencing over 300% growth in the share of the population employed in home care, and some states experiencing growth of less than 30%. This study uses state-level data from a variety of sources to explain this variation in growth across states and to quantify the contribution of different demand- and supply-side factors to the growth in the home care workforce. On the demand side, we consider changes in public financing (Medicaid HCBS spending, Medicare home health and hospice reimbursements), population aging and chronic disease burden, and nursing home capacity. On the supply side, we examine changes in the share of the non-elderly population in a state that is foreign-born, and the share without a college education, as these populations are significantly more likely than average to work in the home care industry. We find that demand-side factors can explain almost all of the variation in growth across states. In particular, the growth of public financing in Medicaid and Medicare–which nationally increased by 222% and 140%, respectively, over this period–can explain the majority of the variation in growth across states. These results have implications for understanding differences in the size of the workforce across states, which may impact older adults and persons with disabilities’ access to paid home care.

